# Wavelet Packet Entropy for Heart Murmurs Classification

**DOI:** 10.1155/2012/327269

**Published:** 2012-11-25

**Authors:** Fatemeh Safara, Shyamala Doraisamy, Azreen Azman, Azrul Jantan, Sri Ranga

**Affiliations:** ^1^Department of Computer Engineering, Islamic Azad University, Islamshahr Branch, Islamshahr, Tehran 3314767653, Iran; ^2^Faculty of Computer Science and Information Technology, 43400 Serdang, Selangor Darul Ehsan, Malaysia; ^3^Department of Cardiology, Serdang Hospital, 43000 Kajang, Selangor Darul Ehsan, Malaysia

## Abstract

Heart murmurs are the first signs of cardiac valve disorders. Several studies have been conducted in recent years to automatically differentiate normal heart sounds, from heart sounds with murmurs using various types of audio features. Entropy was successfully used as a feature to distinguish different heart sounds. In this paper, new entropy was introduced to analyze heart sounds and the feasibility of using this entropy in classification of five types of heart sounds and murmurs was shown. The entropy was previously introduced to analyze mammograms. Four common murmurs were considered including aortic regurgitation, mitral regurgitation, aortic stenosis, and mitral stenosis. Wavelet packet transform was employed for heart sound analysis, and the entropy was calculated for deriving feature vectors. Five types of classification were performed to evaluate the discriminatory power of the generated features. The best results were achieved by BayesNet with 96.94% accuracy. The promising results substantiate the effectiveness of the proposed wavelet packet entropy for heart sounds classification.

## 1. Introduction

Accurate and early diagnosis of cardiac diseases is of great importance which is possible through heart auscultation. It is the most common and widely recommended method to screen for structural abnormalities of the cardiovascular system. Detecting relevant characteristics and forming a diagnosis based on the sounds heard through a stethoscope, however, is a skill that can take years to be acquired and refine. The efficiency and accuracy of diagnosis based on heart sound auscultation can be improved considerably by using digital signal processing techniques to analyze phonocardiographic (PCG) signals [1–3].

Phonocardiography is the recording of sonic vibrations of heart and blood circulation. PCG signals can provide valuable information regarding the performance of heart valves, therefore it has a high potential for detecting various heart diseases [4, 5]. 

**Figure 1:**
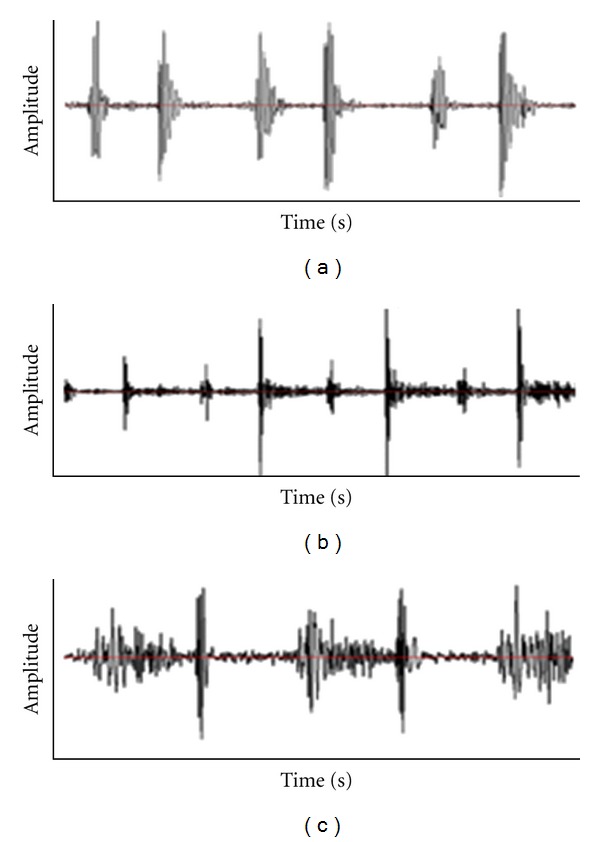
Three samples of heart sounds and murmurs: (a) normal heart sound, (b) aortic regurgitation, and (c) aortic stenosis.

Two loudest heart sounds are the first and the second sounds, referred to as S1 and S2. The time interval between S1 and S2 is called systole and the time interval between S2 and next S1 is called diastole. Normal heart sounds are low-frequency transient signals produced by the heart valves while pathological heart sounds, such as heart murmurs, are high-frequency, noise-like sounds [6]. Heart murmurs are produced as a result of turbulence in blood flow through narrow cardiac valves or reflow through the atrioventricular valves. Congenital heart defects or acquired heart valve diseases are often the cause of abnormal heart murmurs. Aortic stenosis, mitral regurgitation, aortic regurgitation, and mitral stenosis are among the most common pathological types of murmurs. These categories of murmurs were considered in this paper to be distinguished from normal heart sounds.  Figure 1 illustrates the phonocardiogram of one normal and two samples of pathological heart sounds.

Research on PCG signals utilizing signal processing techniques is on the rise because of the ability of PCG recordings to represent important characteristics of the heart sounds. A large number of these studies have been focused on investigating the possibility of the PCG signal classification towards the diagnosis of heart valve disorders [7–11]. 

In general, two major processes are needed prior to actual classification, signal analysis and feature generation. Different signal analysis techniques are employed in the literature such as discrete Fourier transform (DFT), short time Fourier transform (STFT), Wigner distribution, Hilbert transform, continuous wavelet transform (CWT), discrete wavelet transform (DWT), and wavelet packet transform (WPT). Of these, the Fourier and wavelet family of transforms have been more widely used for PCG signal analysis. However, with medical applications, wavelet transform has been found to be one of the best transform for analyzing transient and nonstationary signals, such as PCG [3]. This is because the wavelet provides a reasonable resolution in both the time and frequency domain. Therefore, in this paper, WPT is used to analyze PCG signals. This is further discussed in Section 2.

Feature generation has a crucial role in obtaining high classification accuracies. A number of features based on wavelet transforms have been defined in recent studies to classify different types of heart sounds and murmurs. For instance, Ahlstrom et al. [12] extracted energy and entropy from DWT of the PCG signals in order to classify systolic murmurs. A series of studies were also conducted by Choi et al. [3, 13, 14] whereby new features based on the wavelet transform were defined, such as the normalized autoregressive power spectral density curve, the mean of wavelet packet energy, standard deviation of wavelet packet energy, the maximum peak frequency, the position index of the wavelet packet coefficient corresponding to the maximum peak frequency, and the ratios of the wavelet energy and entropy information.

 In our preliminary study [15], temporal, spectral, and geometric features were combined for heart sound classification. The feature set included zero-crossing rates as the temporal feature, and spectral roll-off, spectral energy entropy, spectral flux, and spectral centroid as the spectral features. Geometric features were added to this feature set, namely, summation of the first order derivatives, summation of the second order derivatives, curve length, area under curve, and centralized mean square values. 

It is clear from these studies that entropy and features defined on entropy have been successfully utilized for heart sound classification. New entropies have been also defined and used in biomedical signal analysis. In the study by Vitulano and Casanova [16], entropy was introduced as a feature for analyzing one-dimensional signal. The entropy was measured as the ratio between signal perturbation and the total signal energy. The experimentation carried out on mammographic signals with different pathologies. Linear transformation has been performed to transform 2D mammographic signal into 1D signal. This entropy, introduced by Vitulano and Casanova [16], is adopted for this study to generate discriminative features from PCG signals. This has not been previously examined for heart sound classification. 

Heart sound classification performed by most studies included classifying normal heart sound and murmurs. Several studies further investigated classifying various categories of murmurs. Neural network is a powerful classifier that was widely used in the past. Ahlstrom et al. [12] used a feed-forward neural network to evaluate a large number of linear and nonlinear features proposed based on DWT and achieved 86% accuracy in classifying systolic murmurs. Neural network was also used in another work by Ahlstrom et al. [17] to differentiate innocent murmurs from aortic stenosis, and a sensitivity of 90% and a specificity of 88% were obtained. Babaei and Geranmayeh [18] utilized a multilayer perceptron neural network (MLP) to investigate the potential of the main statistical characteristics of PCG signals for distinguishing murmurs and acquired 94.24% accuracy for classifying AR, AS, and PS (pulmonary stenosis).

Support vector machine (SVM) is another classifier that was commonly used. Two studies by Choi et al. [3, 14] were reported based on WPT and SVM classifier. In the former study, normal heart sounds were distinguished from murmurs with 96% sensitivity and 100% specificity [14]. In the later one, normal heart sounds were differentiated from regurgitation types of murmurs with 99.78% specificity and 99.43% sensitivity [3]. Choi and Jiang [13] also utilized autoregressive power spectral analysis and multisupport vector machine for PCG signals classification and achieved 99.5% sensitivity and 99.9% specificity to classify normal heart sounds from pathological ones. In addition to MLP and SVM that have been widely used for heart sound classification, KNN, BayesNet, and decision tree classifiers were included to evaluate the discriminatory power of the generated features.

The rest of this paper is organized as follows. In Section 2, the background theory of WPT and the entropy is provided. Details of processes followed for heart sound classification are explained in Section 3. Experiments including data collection and results of classification through generated features are discussed in Section 4. Final conclusions and potential extensions are given in Section 5.

## 2. Wavelet Packet Entropy

Wavelet transform is a powerful technique in analyzing nonstationary signals such as PCG signals [6]. The main advantage of wavelet transform is its varying window size that is narrow for high frequencies and wide for low frequencies. Therefore, wavelet transform is much more powerful than the other time frequency analysis techniques such as DFT and STFT, not only for providing useful time and frequency information, but also for its adaptive time and frequency resolution [19]. 

Selecting appropriate wavelet transform for a given application is also important. WPT was exploited in this paper, instead of CWT and DWT that were widely used in the past studies. A comparison between DWT and WPT by Debbal and Bereksi-Reguig [20] showed that DWT was more suitable than WPT in filtering of clicks and murmurs while WPT provides comprehensive information for a better understanding of the time-frequency characteristics of the cardiac sound. 

One of the quantitative measures associated with WPT is entropy. Entropy provides valuable information for analyzing nonstationary signals. The background theory of WPT and the definition of the entropy used in the current study are explained in this section.

### 2.1. Wavelet Packet Transform

**Figure 2:**
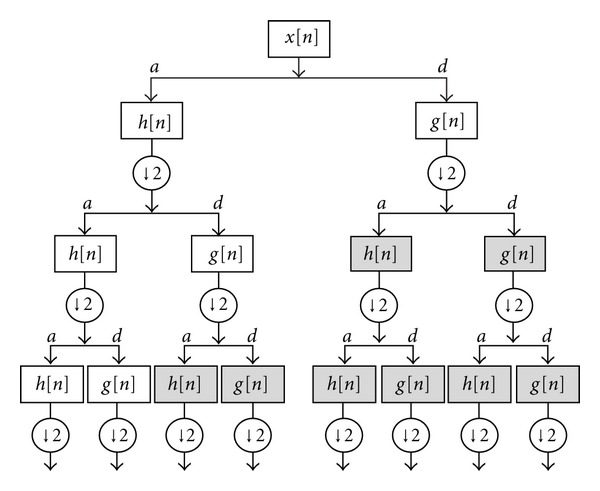
Wavelet packet tree with corresponding high-pass and low-pass filters (*a* = approximation coefficients, *d* = detail coefficients). The shaded nodes indicate the node not to be produced by DWT.

WPT is an extension of DWT whereby all nodes in the tree structure are allowed to split further at each level of decomposition. With WPT, both the approximation and detail coefficients are decomposed into approximation and detail components, in comparison to DWT that decomposes only the approximation coefficients of the signal as shown in Figure 2. Therefore, features can be generated based on approximation and detail coefficients at different levels to obtain more information. The WPT of a signal *x*(*t*) is defined as follows:

(1) $$\begin{array}{c} x_{p}^{n,j}=2^{j/2}\int_{R}^{}{x\left(t\right)\psi }_{n}\left(2^{-j}t-p\right)dt,\;0\leq n\leq 2^{S}-1, \end{array}$$

where *n* is the channel number, *j* is the number of decomposition level, or scale parameter, *p* is the position parameter, *ψ*_*n*_(*t*) is the mother wavelet, and *S* is the maximum decomposition level. After decomposing signal *x*(*t*) by WPT, 2^*S*^ sequences can be produced in the *S*th level. The fast decomposition equation for this kind of WPT is

(2) $$\begin{array}{c} x_{k}^{2n,j+1}=\sum_{p\in Z}h\left(p-2k\right)x_{p}^{n,j},\\ x_{k}^{2n+1,j+1}=\sum_{p\in Z}g\left(p-2k\right)x_{p}^{n,j}, \end{array}$$

where *h*(*i*) and *g*(*i*) are wavelet quadrature mirror filter coefficients.

Three levels of the wavelet packet decomposition with the high-pass and low-pass filters were illustrated in Figure 2. This structure can be continued further to decompose the following approximations and details to reach to a proper level for representing PCG signals of desired murmurs. From the literature, it can be concluded that levels 6 to 8 were generally chosen for analyzing PCG signals of different pathological heart sounds [3, 12, 14, 20, 21].

### 2.2. Entropy

Different types of entropy such as log, norm, Shannon, sure, and threshold can be used to characterize the heart sounds. However, for this study the entropy introduced by Vitulano and Casanova [16] for analyzing 1D signals was utilized. They have transformed the 2D mammographic signal into 1D signal through linear transformation and then applied the entropy on the 1D signal to generate features for differentiating mammograms with different pathologies. They did not utilize any signal processing technique to analyze the signal prior to extract entropy features from the signal. In the current study, the PCG signals were first analyzed with WPT and then entropy features were generated from the wavelet packet coefficients.

Vitulano and Casanova [16] defined the signal “crest” as the part embraced between lines parallel to the abscissas axis, in which the ordinates are *m* and *M*, *m* is the absolute minimum and *M* is the absolute maximum of the signal. Therefore, the signal crest included all the points *x*(*t*) ∈ *X*(*t*), so that

(3) $$\begin{array}{c} m\leq x\left(t\right)\leq M, \end{array}$$

and crest energy is defined as

(4) $$\begin{array}{c} E_{c}=\sum_{i=m}^{M}x_{i},\;i\equiv \left[m,M\right]. \end{array}$$

Signal entropy can be defined based on *E*_*c*_ as

(5) $$\begin{array}{c} S=1-\frac{E-E_{c}}{E},\;S\equiv \left[0,1\right], \end{array}$$

where *E* is signal energy, *E*_*c*_ is crest energy, and *S* is signal entropy. 

Signal entropy *S* is defined based on one-dimensional signals and it has a potential to be applied on the other dimensional signals such as PCG signals.

## 3. Methodology and Materials

Classification requires a sequence of processes to be performed, including preprocessing, signal analysis, feature generation, and classification. Each process is discussed for heart murmur classification in this study.

### 3.1. Preprocessing

The preprocessing of PCG signals carried out in this study includes resampling, filtering, normalization and segmentation. 

#### 3.1.1. Resampling

Feature generation algorithms are highly dependent on the frequency sampling of the electronic stethoscope. Sampling frequencies 4, 5, 10, 20, 25, 40, and 50 kHz can be seen among the heart sounds obtained from online resources and sounds available in the market as auscultation training CDs. In order to remove the heterogeneity of the collected PCG signals, the original signals in the core frequency sampling were mapped into a new 4 kHz. The signal was considered as a time series and new samples are produced by application of the truncated sinc function interpolation.

#### 3.1.2. Filtering

To remove noise from PCG signals, a bandpass finite-duration impulse response (FIR) filter was adopted with preliminary testing. Kaiser window with cut-off frequencies of 25 and 700 Hz was used because heart sound signals are in the range less than 700 Hz [2, 14, 21]. The frequency range of 49–51 was also removed to eliminate the power line noise.

#### 3.1.3. Normalization

The amplitude of heart sounds depend on the pressure on skin measured by electronic stethoscope as well as the setting of the amplifier of the stethoscope. Equation (6), a commonly used normalization equation, was used in this paper to reduce amplitude variations as

(6) $$\begin{array}{c} x_{\mathrm{norm}}\left(n\right)=\;\frac{x\left(n\right)}{\max\left(\left|x\left(n\right)\right|\right)}, \end{array}$$

where *n* number of data points, *x*(*n*) is the PCG signal, and *x*_norm⁡_(*n*) is the normalized signal to be used in this work.

#### 3.1.4. Segmentation

PCG recordings were segmented into their systoles and diastoles. The exact onset and offset locations of each systole and diastole were determined manually under the supervision of a cardiologist. 

### 3.2. PCG Signal Analysis

Following the discussion in Section 2, WPT was utilized for PCG signal analysis. A wavelet packet tree was constructed and the coefficients were calculated for terminal nodes of the tree. The appropriate decomposition level was determined based on the following equation:

(7) $$\begin{array}{c} R=\frac{0.5*\text{FS}}{2^{\text{DL}}}, \end{array}$$

where *R* is the resolution, 0.5 is the Nyquist coefficient, FS is the sampling frequency, and DL is the suitable decomposition level with desired resolution. Since PCG signals were resampled into 4 KHz, resolution of 31.25 Hz would be achieved at level six using (7) that is a reasonable resolution for this study.

After determining the level of decomposition, four types of mother wavelets were examined as potential mother wavelets: The Mayer, Symlets, Coiflets and Daubiches. This was performed by computing the error existing between the original signal and the synthesis signal (i.e., wavelet packet transform of the signal). The error was calculated by the following equation that was defined by Cherif et al. [2]:

(8) $$\begin{array}{c} E_{\text{error}}=\frac{\sum_{i=1}^{N}{\left|S_{oi}-S_{ri}\right|}_{}}{N}, \end{array}$$

where *S*_*o*_ is the original signal, *S*_*oi*_ is the sample of *S*_*o*_, *S*_*r*_ is the synthesised signal, and *S*_*ri*_ is the sample of *S*_*r*_. Using the above equation, the lowest error was obtained for Daubiches which was chosen as the mother wavelet for analysing the PCG recordings. 

### 3.3. Feature Generation

Features are representatives of the underlying signal and proper choice of features results in higher classification accuracy. As stated in Section 1, entropy was successfully used as a feature for heart sound classification. In this work also the entropy was utilized to generate discriminative features from PCG recordings. However, the entropy introduced by Vitulano and Casanova [16] (explained in Section 2.2) was exploited, instead of common entropies such as Shannon and log [3, 12, 22]. 

Systole and diastole segments of each PCG signals were represented in the sixth decomposition level of WPT with Daubiches8, as described in the previous section. There are 2^6^ terminal nodes in the 6th decomposition level. The entropy was computed from the coefficients of all terminal nodes and 64 entropies were obtained for each segment. Then a feature vector including all 128 entropies was constructed. In order to reduce the dimensionality of the feature space, principal component analysis (PCA) was applied to feature vectors. Number of features to be selected was determined by trial and error. Thirty two features were chosen from each feature vector to be fed into classifier.

### 3.4. Classification

The discriminatory power of the generated features was evaluated via KNN, BayesNet, and decision tree classifiers in addition to SVM and MLP that were widely used in previous studies.

## 4. Experiments

The classification accuracy using wavelet packet entropy was evaluated with a collection of 350 heart sounds as discussed in the following section. A standard data collection for PCG signal is not available and the PCG signals from various sources were collected.

### 4.1. Data Collection

A data set of 350 heart sounds comprising 50 normal and 300 murmurs was employed. For murmurs, 80 MR, 100 AS, 50 AR, and 70 MS were obtained. Pathological heart sounds were collected from auscultation training materials where the sound categories were explicitly specified. Evaluation was performed based on the annotations provided. For the normal category, sounds were recorded using Welch-Allyn Meditron electronic stethoscope. 

### 4.2. Results

**Figure 3:**
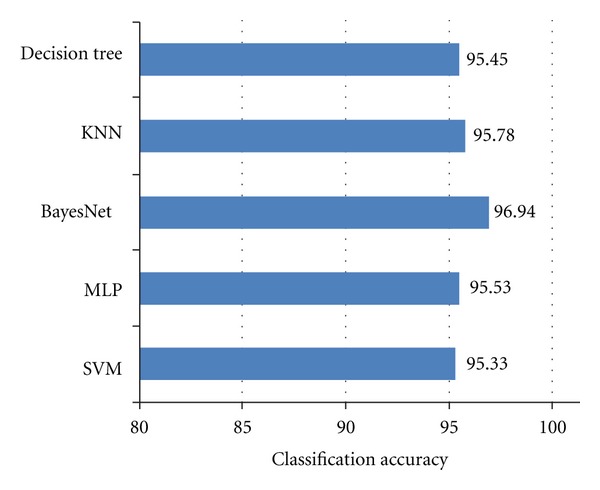
Accuracy of PCG signals classification using the wavelet packet entropy.

Classification accuracy reflects the potential strength of generated features to differentiate underlying signals from each other. For each of the 350 heart sounds prepared for this study, a feature vector comprising thirty-two features was generated (Section 3.3) and classified. The classification accuracy was determined using a 10-fold cross-validation. The classification accuracy of each classifier is presented in Figure 3. Although the accuracies cannot be benchmarked against other studies due to the lack of standard data collections, the accuracies obtained following the experiments in this study are quite high showing the feasibility of using wavelet packet entropy for heart sound classification. The best and worst accuracies were achieved by BayesNet and SVM, with 96.94% and 95.33%, respectively.

Despite the small difference between the accuracy of classifiers, the results of the classification establishes the feasibility of entropy introduced by Vitulano and Casanova [16] for classifying PCG signals.

## 5. Conclusion

The feasibility of using the entropy for heart murmur classification was shown in this paper. The entropy was previously introduced for analyzing mammographic signals. It was defined as the ratio between signal perturbation and the total signal energy. In this study, the entropy was calculated from wavelet packet coefficients of PCG recordings as a feature to be used for classifying heart sounds. High classification accuracy of 96.94% is achieved by BayesNet classifier that indicates the viability of the entropy as a feature to describe PCG signals. A more extensive comparative study would be required to evaluate the proposed features against other features introduced for PCG signal classification on the same PCG database. In this study, normal heart sounds were differentiated from four types of murmurs. The heart sound categories would be expanded to include different murmurs.

## References

[1] Chen Y, Wang S, Shen C-H, Choy F (2012). Intelligent identification of childhood musical murmurs. *Journal of Healthcare Engineering*.

[2] Cherif LH, Debbal SM, Bereksi-Reguig F (2010). Choice of the wavelet analyzing in the phonocardiogram signal analysis using the discrete and the packet wavelet transform. *Expert Systems with Applications*.

[3] Choi S, Shin Y, Park HK (2011). Selection of wavelet packet measures for insufficiency murmur identification. *Expert Systems with Applications*.

[4] Gupta CN, Palaniappan R, Swaminathan S, Krishnan SM (2007). Neural network classification of homomorphic segmented heart sounds. *Applied Soft Computing Journal*.

[5] Jia L, Song D, Tao L, Lu Y (2012). Heart sounds classification with a fuzzy neural network method with structure learning. *Advances in Neural Networks*.

[6] Ergen B, Tatar Y, Gulcur HO (2011). Time-frequency analysis of phonocardiogram signals using wavelet transform: a comparative study. *Computer Methods in Biomechanics and Biomedical Engineering*.

[7] Kao WC, Wei CC (2011). Automatic phonocardiograph signal analysis for detecting heart valve disorders. *Expert Systems with Applications*.

[8] Salama M, Hassanien A, Platos J, Fahmy A, Snasel V Rough sets-based identification of heart valve diseases using heart sounds.

[9] Sanei S, Ghodsi M, Hassani H (2011). An adaptive singular spectrum analysis approach to murmur detection from heart sounds. *Medical Engineering & Physics*.

[10] Tseng YL, Ko PY, Jaw FS (2012). Detection of the third and fourth heart sounds using Hilbert-Huang transform. *BioMedical Engineering OnLine*.

[11] Yuenyong S, Nishihara A, Kongprawechnon W, Tungpimolrut K (2011). A framework for automatic heart sound analysis without segmentation. *BioMedical Engineering Online*.

[12] Ahlstrom C, Hult P, Rask P (2006). Feature extraction for systolic heart murmur classification. *Annals of Biomedical Engineering*.

[13] Choi S, Jiang Z (2010). Cardiac sound murmurs classification with autoregressive spectral analysis and multi-support vector machine technique. *Computers in Biology and Medicine*.

[14] Choi S (2008). Detection of valvular heart disorders using wavelet packet decomposition and support vector machine. *Expert Systems with Applications*.

[15] Safara F, Doraisamy S, Azman A, Jantan A (2012). Heart sounds clustering using a combination of temporal, spectral and geometric 8 features. *Computing in Cardiology*.

[16] Vitulano S, Casanova A (2008). The role of entropy: mammogram analysis. *Image Analysis and Recognition*.

[17] Ahlstrom C, Höglund K, Hult P, Häggström J, Kvart C, Ask P Distinguishing innocent murmurs from murmurs caused by aortic stenosis by recurrence quantification analysis.

[18] Babaei S, Geranmayeh A (2009). Heart sound reproduction based on neural network classification of cardiac valve disorders using wavelet transforms of PCG signals. *Computers in Biology and Medicine*.

[19] Chen Y, Wang S, Shen C-H, Choy FK (2012). Matrix decomposition based feature extraction for murmur classification. *Medical Engineering & Physics*.

[20] Debbal SM, Bereksi-Reguig F (2007). Time-frequency analysis of the first and the second heartbeat sounds. *Applied Mathematics and Computation*.

[21] Homaeinezhad MR, Atyabi SA, Deneshvar E, Ghaffari A, Tahmasebi M (2011). Optimal delineation of PCG sounds via false-alarm bounded segmentation of a wavelet-based principal components analyzed metric. *International Journal for Numerical Methods in Biomedical Engineering*.

[22] Sengur A, Turkoglu I (2008). A hybrid method based on artificial immune system and fuzzy k-NN algorithm for diagnosis of heart valve diseases. *Expert Systems with Applications*.

